# The activating *TERT* promoter mutation C228T is recurrent in subsets of adrenal tumors

**DOI:** 10.1530/ERC-14-0016

**Published:** 2014-06

**Authors:** Tiantian Liu, Taylor C Brown, C Christofer Juhlin, Adam Andreasson, Na Wang, Martin Bäckdahl, James M Healy, Manju L Prasad, Reju Korah, Tobias Carling, Dawei Xu, Catharina Larsson

**Affiliations:** 1 Department of Medicine-Solna, Karolinska Institutet, Karolinska University Hospital CMMStockholm, SE-171 76Sweden; 1 Yale Endocrine Neoplasia Laboratory, Yale School of Medicine333 Cedar Street, FMB130A, PO Box 208062, New Haven, Connecticut, 06520USA; 2 Department of Surgery, Yale School of MedicineNew Haven, ConnecticutUSA; 3 Department of Oncology-Pathology, Karolinska Institutet, Karolinska University Hospital CCKStockholm, SE-171 76Sweden; 4 Department of Molecular Medicine and Surgery, Karolinska Institutet, Karolinska University HospitalStockholm, SE-171 76Sweden; 5 Department of Pathology, Yale School of MedicineNew Haven, ConnecticutUSA

**Keywords:** *TERT*, telomerase, mutation, endocrine, pheochromocytoma, adrenocortical tumor

## Abstract

The telomerase reverse transcriptase gene (*TERT*) encodes the reverse transcriptase component of the telomerase complex, which is essential for telomere stabilization and cell immortalization. Recent studies have demonstrated a transcriptional activation role for the *TERT* promoter mutations C228T and C250T in many human cancers, as well as a role in aggressive disease with potential clinical applications. Although telomerase activation is known in adrenal tumors, the underlying mechanisms are not established. We assessed C228T and C250T *TERT* mutations by direct Sanger sequencing in tumors of the adrenal gland, and further evaluated potential associations with clinical parameters and telomerase activation. A total of 199 tumors were evaluated, including 34 adrenocortical carcinomas (ACC), 47 adrenocortical adenomas (ACA), 105 pheochromocytomas (PCC; ten malignant and 95 benign), and 13 abdominal paragangliomas (PGL; nine malignant and four benign). *TERT* expression levels were determined by quantitative RT-PCR. The C228T mutation was detected in 4/34 ACCs (12%), but not in any ACA (*P*=0.028). C228T was also observed in one benign PCC and in one metastatic PGL. The C250T mutation was not observed in any case. In the ACC and PGL groups, *TERT* mutation-positive cases exhibited *TERT* expression, indicating telomerase activation; however, since expression was also revealed in *TERT* WT cases, this could denote additional mechanisms of *TERT* activation. To conclude, the *TERT* promoter mutation C228T is a recurrent event associated with *TERT* expression in ACCs, but rarely occurs in PGL and PCC. The involvement of the *TERT* gene in ACC represents a novel mutated gene in this entity.

## Introduction

**Figure 1:**
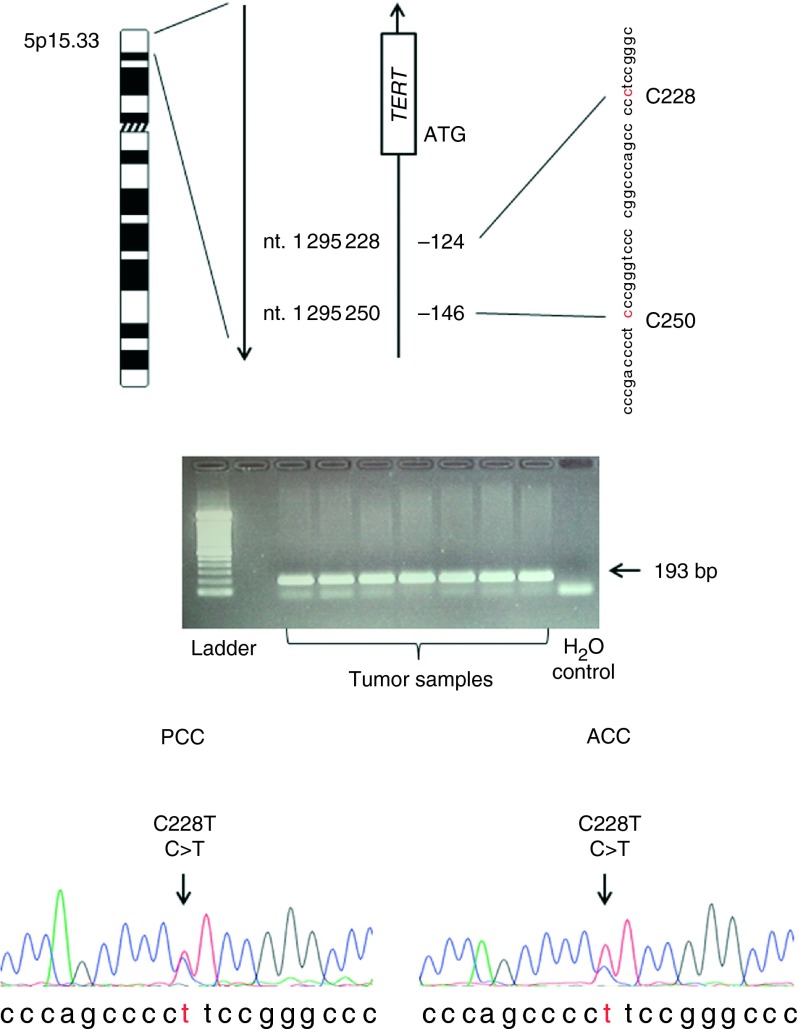
Detection of *TERT* promoter mutations. (Top) Location of the commonly mutated nucleotides C228 and C250 in relation to the *TERT* promoter positions and the genomic positions within chromosomal region 5p15.33. (Middle) 1% Agarose gel displaying the 193 bp amplicon of the *TERT* promoter. The smaller band represents unspecific primer complex formations. (Bottom) Sanger sequencing chromatograms displaying heterozygous C228T *TERT* promoter mutations in a pheochromocytoma (PCC) and an adrenocortical carcinoma (ACC).

Telomerase, an RNA-dependent DNA polymerase, synthesizes telomeric TTAGGG repeats essential for chromosome stability (Blackburn 2010). Normal human differentiated cells in general lack telomerase activity, and thus exhibit a progressive decline in telomere length with each cell division due to the ‘end-replication problem’. Very short telomeres are unable to maintain their functional structure and thereby activate the DNA damage response pathway through which apoptosis or senescence is induced. Therefore, telomere shortening prevents infinite cellular proliferation. In contrast, unlimited proliferation is a hallmark of malignant cells. During the oncogenic process, stabilization of telomere length is required to acquire an unlimited proliferation potential, and is achieved by telomerase activation in the majority of human malignancies (Cong *et al*. 2002). Multiple factors or pathways contribute to the cancer-specific telomerase activation, but the primary determinant is telomerase reverse transcriptase (TERT), a catalytic subunit of the telomerase enzyme complex. As the de-repression of *TERT* gene transcription is intimately coupled with the acquisition of telomerase activity in transformed cells, regulatory mechanisms controlling *TERT* transcription have been extensively investigated in recent years (Eldholm *et al*. 2013, Wu *et al*. 2013, Xu *et al*. 2013). Despite repeated efforts, it remains incompletely understood exactly how the *TERT* transcription is activated in carcinogenesis. Recently, *TERT* promoter mutations have been reported in human malignancies, which create *de novo* ETS1-binding motifs stimulating the *TERT* transcription. This genetic event is thus proposed as a novel mechanism activating telomerase in malignant cells. The two recurrent mutations C228T and C250T (Fig. 1) were first described in melanoma (Horn *et al*. 2013, Huang *et al*. 2013), and have since been detected in a variety of human tumors (Allory *et al*. 2013, Killela *et al*. 2013, Nonoguchi *et al*. 2013, Remke *et al*. 2013, Tallet *et al*. 2013, Vinagre *et al*. 2013, Zhao *et al*. 2013). In thyroid carcinomas the mutation is detected in aggressive forms and has prognostic significance (Landa *et al*. 2013, Liu *et al*. 2013*a*,*b*).

Pheochromocytomas (PCC) and abdominal paragangliomas (PGL) are typically catecholamine-producing tumors, originating from chromaffin cells in the paraganglia inside or outside the adrenal medulla (Elder *et al*. 2005). Adrenocortical carcinoma (ACC) and adenoma (ACA) may secrete, e.g. cortisol, aldosterone, or androgens or may be hormonally non-functional. Telomerase activation and *TERT* expression are frequently seen in ACC and malignant PCC/PGL, but not in ACA or benign PCC/PGL (Elder *et al*. 2003, Else *et al*. 2008). However, it is currently unclear whether this upregulation is coupled to *TERT* promoter mutations. Since little is known regarding the *TERT* promoter mutational status in adrenocortical and adrenomedullary tumors, we sought to determine the mutational status of the *TERT* promoter and explore potential associations between this genetic event and tumor phenotypes as well as patient outcome for these tumor types.

## Subjects and methods

### Study cohort

We analyzed a total of 199 tumors from 199 patients operated upon at Karolinska University Hospital, Stockholm, Sweden (Series A) or Yale University School of Medicine, New Haven USA (Series B). All tumors constituted primary tumor tissue except for one ACC specimen (ACC-34) that was derived from a recurrent operation in the adrenal fossa. Informed consent was obtained from patients before surgical resection of tumor tissue according to protocols approved by the local Ethical committee of Karolinska Institutet, Stockholm Sweden and the Institutional Review Board at Yale University, CT, USA, respectively. In addition, one case of adrenal hyperplasia, one adrenal cyst, and three non-cancerous normal adrenal tissue samples were included. Clinical details for subsets of the tumor cases have been previously published (Andreasson *et al*. 2013*a*,*b*, Caramuta *et al*. 2013).

Tumor classifications were based on the routine histopathological examinations and followed the guidelines of the World Health Organization (WHO; DeLellis *et al*. 2004). For PCC and PGL cases, the criteria of the Armed Forces Institute of Pathology AFIP were applied for identification of malignancy (Lack 2007). Thus the 199 tumors included 34 ACCs, 47 ACAs, 105 PCCs (ten malignant and 95 benign), and 13 PGLs (nine malignant and four benign).

Clinical information and follow-up for the ACC cases are detailed in Supplementary Table S1, see section on supplementary data given at the end of this article. The ACA group included 35 female and 12 male patients with a median age of 52 years at diagnosis (mean 51, range 16–79) and the tumors were biochemically classified as aldosterone-producing (*n*=30), cortisol-producing (*n*=10), or non-hyperfunctioning (*n*=7 including one unknown). Recurrences were not detected in any of the ACA patients for which follow-up were available. The PCC group constituted 63 female and 42 male patients with a median age of 55 years at diagnosis (mean 55 years, range 19–84). Ten of the PCCs were classified as malignant based on AFIP criteria, one of which also showed metastasis at the time of diagnosis according to the WHO criteria for malignant PCC. The PGL group (Supplementary Table S2) included nine male and four female patients with a median age at diagnosis of 41 years (mean 41 years, range 14–73). Nine PGLs were classified as malignant according to AFIP, four of which also exhibited metastases at diagnosis or follow-up in agreement with the WHO criteria for malignancy.

### DNA and RNA extractions

Genomic DNA (gDNA) was extracted from fresh frozen tissue samples using the DNeasy Blood and Tissue DNA isolation kit (Qiagen) or the AllPrep Mini Kit (Qiagen) following the protocols of the manufacturer. One ACC (ACC-32) was extracted from the formalin-fixated paraffin-embedded (FFPE) tumor tissues using an internal protocol at the Keck DNA Sequencing Facility, Yale University. The FFPE tissue was examined and representative regions were marked for coring by an experienced endocrine pathologist. Amount and quality of DNA were measured by a NanoDrop (Thermo Fisher Scientific, Waltham, MA, USA). Total RNA was extracted from 27 ACCs from Series A (ACC 1–27), 12 PGLs from series A (PGL 1–3 and PGL 5–13), and from 63 PCCs in Series A using the *mir*Vana miRNA Isolation Kit (Ambion, Austin, TX, USA) and analyzed in an Agilent 2100 Bioanalyzer (Agilent, Santa Clara, CA, USA). All cases were well preserved and RNA preparations displayed RIN values >7 as reported (Andreasson *et al*. 2013*a*,*b*).

### *TERT* promoter sequencing

The *TERT* promoter region was sequenced for detection of the two mutations C228T and C250T using previously applied methodology (Liu *et al*. 2013*a*). The target region was amplified by conventional PCR using the previously reported primers 5′-CACCCGTCCTGCCCCTTCACCTT-3′ (sense, binding to the coding strand) and 5′-GGCTTCCCACGTGCGCAGCAGGA-3′ (antisense, binding to the complementary strand). The PCR was run in 25 μl reactions with 7.5 μl betaine (5 M), 4 μl dNTPs (1.25 mM), 2.5 μl 10×PCR buffer, 2.5 μl MgCl_2_ (25 mM), 10 μM of each primer, 0.125 μl Taq polymerase (5U)/μl (HotStar Taq DNA Polymerase Qiagen (Cat# 203205) or Platinum Taq DNA polymerase High Fidelity, Invitrogen by Life Technologies (Cat# 11304-011)), and 2 μl gDNA. The amount of the gDNA for each PCR varied from 20 ng to ∼400 ng depending on the quality of the sample. PCR conditions consisted of one cycle of 95 °C for 8 min and 62 °C for 2 min, followed by 40 cycles of (72 °C for 2 min 30 s, 95 °C for 15 s, 62 °C for 1 min), and finally elongation at 72 °C for 7 min.

Amplicons were visualized in 1 or 2% agarose gels and verified to have the expected size of 193 bp (Fig. 1). *TERT* promoter sequences were generated by Sanger sequencing of amplicons using an Applied Biosystems 3730 capillary instrument and fluorescently labeled dideoxynucleotides (Big Dye Terminator, Life Technologies). Sequencing was carried out in-house (Series A) or at the Keck DNA Sequencing Facility, Yale University (Series B). The samples were sequenced in both directions, and all samples were successfully sequenced using either the sense or antisense primer, or both. All mutations were confirmed with both sense and antisense primers.

### *TERT* expression

*TERT* mRNA expression levels were determined in 27 ACCs and in 75 adrenomedullary tumors (63 PCC and 12 PGL) by quantitative real-time PCR (qRT-PCR). Taqman Gene Expression Assays (Applied Biosystems) were used for the target gene *TERT* (Hs00972656_m1, catalogue no. 4331182) and the house-keeping genes *18S* rRNA (Hs99999901_s1) for ACCs and *beta-2-microglobulin* (*B2M*, NM_004048.2, catalogue no. 4310886E) for PCCs and PGLs, according to previous selections of endogenous controls (Andreasson *et al*. 2013*a*,*b*, Caramuta *et al*. 2013). Reactions were run using standard protocols and an ABI 7900HT Real-time PCR System (Applied Biosystems). *TERT* expression levels were calculated from threshold cycle values and normalization to *18S* and *B2M* values respectively (2^(−ΔCt)^). In a control experiment employed for one of the runs, we used the gastric cancer cell line BGC-823 as a reference, and if the level in a given sample was <0.1% of that in BGC-823 cells, it was classified into a *TERT*-negative category.

### Statistical analyses

Statistical calculations were performed using IBM SPSS Statistics 19 (IBM, Armonk, NY, USA). Differences in the *TERT* promoter mutation frequency between tumors with different clinical parameters were determined using Fisher's exact test. Overall survival and disease-related survival for ACC patients were illustrated by Kaplan–Meier plots, and significance was calculated by log-rank test. *P* values of <0.05 were considered as statistically significant.

## Results

### Detection of the *TERT* promoter mutation C228T

We screened a total of 199 tumors for the activating *TERT* promoter mutations C228T and C250T by Sanger-based technology (Table 1). There were a total of six *TERT* promoter mutations identified in our cohort (Supplementary Tables S1 and S2). As exemplified in Fig. 1, all positive cases carried the C228T mutation, while C250T was not detected. Chromatograms were read manually, and mutational zygosity was determined through the mutated to WT peak relation at position C228. Four mutations were observed among the 34 ACCs screened (12%); three heterozygous (ACCs 7, 12, and 34) and one homozygous (ACC 27). Furthermore, single heterozygous mutations were detected in one of 13 PGLs (8%) and in one of 105 PCCs (1%). No *TERT* mutation was identified in the 47 ACAs, or in the adrenocortical hyperplasia, the adrenal cyst, and the three normal adrenal tissues. All mutation-positive samples were confirmed using both sense and antisense sequencing primers.

### *TERT* mRNA expression in tumors with C228T

**Figure 2:**
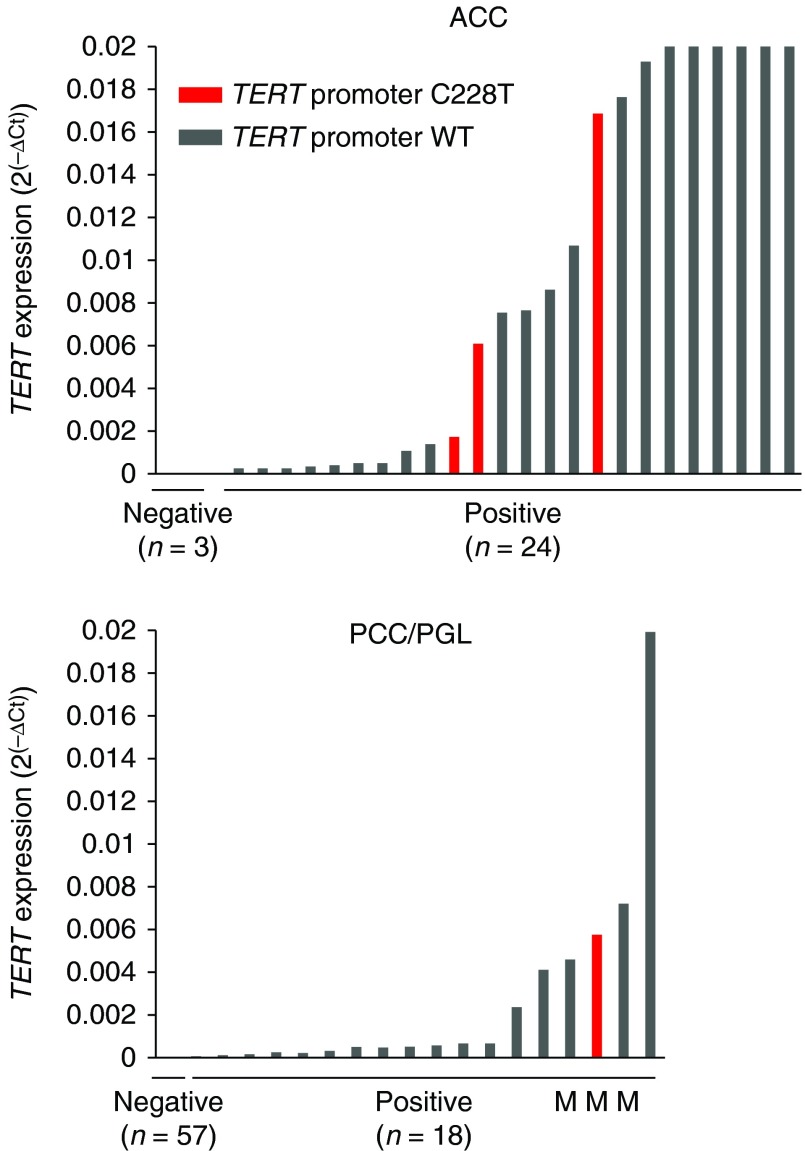
Comparison of *TERT* mRNA expression with *TERT* mutation C228T status. Results from analyses of *TERT* expression in 27 ACCs (top) and in 75 adrenomedullary tumors (PCC and PGL) (bottom). The bars in the diagrams illustrate the *TERT* mRNA expression level (2^(−ΔCt)^) in individual tumors determined by qRT-PCR. *TERT* mutated cases are illustrated in red bars and WT cases in grey bars. For the adrenomedullary tumors, an ‘M’ underneath the corresponding column denotes a malignant tumor as determined by biological evidence of metastatic disease according to the current WHO criteria.

Given that the *TERT* promoter mutation stimulates *TERT* transcription, we quantified *TERT* mRNA expression in a total of 27 ACCs, 63 PCCs, and 12 PGLs from Series A. All three mutation-positive ACCs assayed expressed *TERT* (Fig. 2). Analysis of the remaining mutation-negative tumors revealed that *TERT* was expressed in 21/24 tumors, while in three cases no expression was detectable (Fig. 2). For the PGL group, 3/12 cases (25%) expressed *TERT*, including the sole mutation-positive tumor (Fig. 2; Supplementary Table S2). Among the 63 PCCs, 15 were positive for *TERT* expression (24%) whereas the majority (48/63; 76%) displayed undetectable levels (Fig. 2). No RNA was available to test the only PCC endowed with the C228T mutation.

### Relationship between C228T and molecular phenotypes

Mutation of the succinate dehydrogenase B gene (*SDHB*) is a frequent genetic event in PGL, especially in malignant PGL (Astuti *et al*. 2001, Gimenez-Roqueplo *et al*. 2003). We therefore compared the C228T mutation status with the occurrence of *SDHB* mutations (Kiss *et al*. 2013) in the PGL group. The C228T-positive tumor and three additional PGLs carried a *SDHB* mutation, while nine cases were WT for both gene mutations; however, the limited number of cases does not allow for associations between gene mutations in the PGL entity.

### Relationship between C228T, clinical characteristics, and survival

The *TERT* mutation was significantly associated with the ACC as compared with the ACA phenotype (*P*=0.028, two-tailed Fisher's exact test). However, no significant difference was observed between C228T mutated and WT ACCs, concerning gender, age at diagnosis, overall histology, tumor size, presence of metastasis, or hormonal function. Follow-up of the four mutation positive ACC cases showed that ACC 7 who had metastatic disease at diagnosis died of the disease after 4 months; ACC 34 developed multiple recurrences and died some 5 months after the first surgery; ACC 12 remained disease free but died of other causes after 65 months; and ACC 27 who developed bone metastases is alive after 168 months (Supplementary Table S1). Survival analysis did not reveal a statistically significant difference between the four C228T mutated ACCs and the 30 WT cases (Log Rank=0.191). The PGL patient with a C228T mutated tumor died of the disease after 24 months, while the mutated PCC case was diagnosed as benign but lost to follow-up.

## Discussion

Immortalization is one of the key hallmarks of any given malignant tumor, and increased telomerase activity and *TERT* gene expression are recurrently found in human cancers. In this study, we show for the first time that subsets of adrenocortical and chromaffin cell tumors carry *TERT* promoter mutations, thereby proving a genetic event possibly associated with the upregulation of *TERT* gene expression in these tumor entities.

ACC is a highly heterogeneous tumor type, strongly coupled to dysregulation of the *IGF2* locus, as well as Wnt- and p53-related pathways. The imprinted *IGF2-H19* locus (Larsson 2013) in chromosome 11p15 shows frequent overexpression of the *IGF2* gene and its hosted microRNAs in sporadic ACCs (Gicquel *et al*. 1997, Wilkin *et al*. 2000, Soon *et al*. 2009). In addition, *TP53* and *CTNNB1* mutations are often seen in this tumor type and are functionally linked to tumor progression (Lin *et al*. 1994, Hsu *et al*. 2001, Tissier *et al*. 2005). Even so, a large proportion of sporadic ACCs have not yet been coupled to a specific mutational event. In this study, we establish that the *TERT* promoter mutation C228T is a recurrent event for this tumor entity, with a frequency of 12% in this cohort. Moreover, we observed a statistically significant difference between ACCs and ACAs with regards to the C228T mutation (*P*=0.028). ACCs with the C228T mutation showed *TERT* mRNA expression, demonstrating that the *TERT* mutation leads to telomerase activation in ACC. However, as the majority of *TERT* promoter WT ACCs also exhibited increased *TERT* mRNA expression levels, this may suggest that other genetic and/or epigenetic mechanisms besides the promoter mutational status influence the overall levels of *TERT* gene expression in sporadic ACCs.

From a diagnostic point of view, the specificity of *TERT* promoter mutations in ACCs was 100% given the absence of mutations in the benign counterpart, ACAs. This would in theory make clinical screening for the *TERT* promoter mutation C228T a promising tool to point out malignant cases, even though the sensitivity is poor. However, in our cohort, three out of four ACCs with the C228T mutation were endowed with distant metastases or recurrent disease either at diagnosis or at post-surgical follow-up. Hence these three cases are unequivocally malignant and the responsible pathologists would probably deem a mutational screening pointless for diagnostic purposes in some or all of these cases. One case (ACC 12) constituted a metastatic-free 19-cm ACC, in which the malignant diagnosis was based on the histological findings of a diffuse and highly variable architecture, pleomorphic nuclei, elevated mitotic rate, widespread necrosis as well as multifocal evidence of vascular invasion. Independent studies will be needed to investigate if *TERT* promoter mutational screening in histologically equivocal ACCs would aid in the definite diagnosis.

The *TERT* promoter mutation was found to be a rare event in PCC observed in only 1/105 cases analyzed, which is in agreement with Vinagre *et al*. (2013) who reported lack of *TERT* promoter mutations in 17 cases analyzed. A single mutation in a large cohort of 105 PCC cases would correspond to a frequency of <1%, which would suggest a very minor role in the development in PCC. In a similar manner, only one PGL of 13 (8%) carried a C228T mutation. Given the low number of cases studied, a future study incorporating a larger number of PGLs would help to determine the true prevalence of this mutation type.

*TERT* gene expression was evident in 25% of adrenomedullary tumors assayed (15 PCCs and three PGLs), including the single PGL with a C228T mutation. Given the low burden of *TERT* promoter mutations in adrenomedullary tumors, the detected expression in subsets of PCCs/PGLs with WT sequences suggests that alternate genetic mechanisms are responsible for the observed upregulation of *TERT* mRNA in these cases.

It is particularly interesting to note that *TERT* promoter mutations occur in different types of malignant endocrine tumors, and seem to aggregate in aggressive forms (Landa *et al*. 2013, Liu *et al*. 2013*a*,*b*, Vinagre *et al*. 2013). Although the frequencies vary between cancer types, the 12% prevalence observed here for ACCs is comparable to the findings in papillary thyroid carcinoma (Liu *et al*. 2013*b*). The finding of specific *TERT* promoter mutations in subsets of endocrine cancers could suggest that these tumors share a common molecular denominator regarding the acquisition of cellular immortality. Given this association, we examined whether the single, benign *TERT* mutated PCC had malignant properties not yet visualized on routine histopathological work-up. The initial histopathological examination recognized the lesion as a PCC without unequivocal signs of malignancy according to both WHO and AFIP criteria, and subsequent re-evaluation by an independent expert endocrine pathologist was consistent with a benign diagnosis.

To conclude, *TERT* promoter mutations are predominantly found in the subgroups of malignant endocrine tumors, such as malignant PGL and adrenocortical cancer, and the observation of C228T mutations in 12% of ACCs adds a new cornerstone to the mutational panorama of these tumors. The C228T mutations seem to exhibit low sensitivity in conjunction with high specificity toward biologically aggressive disease; the latter providing a promising platform for further studies assessing *TERT* promoter mutations as a potential malignancy marker in equivocal cases with unknown malignant potential. Moreover, our findings might explain why the subsets of endocrine tumors regularly exhibit high levels of *TERT* gene expression. As the majority of endocrine cancers exhibit high *TERT* levels, additional, unknown genetic or epigenetic, events may explain the observed association between *TERT* gene upregulation and malignant phenotype. Moreover, we did not observe statistically significant associations between C228T mutations and various clinical parameters, including overall survival. Even so, an eventual association would be hard to make given the rather low number of mutated cases studied. Future studies of patients in which long-term data is available will possibly reveal if *TERT* promoter mutations influence survival and if histologically benign PCC with mutation demand more extensive follow-up.

## Supplementary data

This is linked to the online version of the paper at http://dx.doi.org/10.1530/ERC-14-0016.

## Figures and Tables

**Table 1:** Detection of *TERT* promoter mutations in adrenal tumors

**Tumor types and subgroups**	**Number of mutated/analyzed cases** (%)		
Series A (*n*=147)	Series B (*n*=52)	Series A+B (*n*=199)
Adrenocortical tumors	3/48 (6%)	1/33 (3%)	4/81 (5%)
Adrenocortical carcinoma (ACC)	3/27 (11%)	1/7 (14%)	4/34 (12%)
Adrenocortical adenoma (ACA)	0/21	0/26	0/47
Pheochromocytoma (PCC)	0/86	1/19 (5%)	1/105 (1%)
Malignant PCC^a^	0/10	–	0/10
Benign PCC	0/76	1/19 (5%)	1/95 (1%)
Paraganglioma (PGL)	1/13 (8%)	–	1/13 (8%)
Malignant PGL^a^	1/9 (11%)	–	1/9 (11%)
Benign PGL	0/4	–	0/4

a According to the AFIP criteria (Lack 2007).
